# Evaluation of the Oxidative Process of Chia Seed Oil by Means of ESR Combined with LF-NMR and SAXS

**DOI:** 10.3390/foods14244280

**Published:** 2025-12-12

**Authors:** Yun Ma, Nan Wu, Cheng Yang, Fei Liu

**Affiliations:** 1 State Key Laboratory of Food Science and Resources, Jiangnan University, Wuxi 214122, China; yunma@jiangnan.edu.cn (Y.M.); fliua15c@163.com (N.W.); chengyang1861@jiangnan.edu.cn (C.Y.); 2 School of Food Science and Technology, Jiangnan University, Wuxi 214122, China; 3 Science Center for Future Foods, Jiangnan University, Wuxi 214122, China

**Keywords:** electron spin resonance, low-field nuclear magnetic resonance, small-angle X-ray scattering, free radicals, chia seed oil, oxidative stability

## Abstract

Chia seed oil, valued for its health-promoting omega-3 and omega-6 fatty acids, is highly susceptible to oxidation. This study employed a multi-technique approach based on electron spin resonance (ESR), low-field nuclear magnetic resonance (LF-NMR), and small-angle X-ray scattering (SAXS) to monitor its oxidative process. ESR identified alkyl (DMPO-•R) and peroxyl (DMPO-•OOR) radicals as primary species derived from unsaturated fatty acids. This was accompanied by a decrease in relaxation time of peak T_21_, T_22_, and T_23_, and the peak area of S_21_ gradually increased as the heating time increased. The SAXS intensity of chia seed oil at q = 3.4 nm^−1^ increased markedly after heating for 20 h, and the peak shifted to the low q-region with Δq = 0.6 nm^−1^, confirming the significant formation of nanoscale aggregates, which correlated with observed increases in oil turbidity. Our findings demonstrate the value of an integrated analytical strategy for a comprehensive understanding of oxidation in chia seed oil.

## 1. Introduction

Nowadays, oils rich in omega-3 polyunsaturated fatty acids (ω-3 PUFAs) are increasingly used as functional ingredients in healthy foods, dietary supplements, and pharmaceutical products due to their nutritional and health benefits. Over the last few decades, studies have proven that these essential fatty acids are beneficial for the prevention/treatment of obesity, metabolic syndrome, and cardiovascular diseases [1,2,3]. Now these PUFAs are mostly consumed from oils derived from fatty fish, and the main problem that comes is the fishy taste, together with the rising concerns about contamination of fish oil with mercury and other organic pollutants [4]. Also, the intake of these PUFAs does not meet the recommendations about the ω-6:ω-3 ratio of 5–10:1 suggested by international health authorities in most countries worldwide [5], intensifying the investigation regarding the incorporation of ω-3 PUFAs-rich oils into foods. As a consequence, plant oils with a high amount of PUFAs are attracting increased attention as a substitute for fish oils.

Chia seed oil is one of the plant oils that can be obtained from chia seeds through different extraction methods, including cold pressing, solvent extraction, vacuum steam distillation, and supercritical fluid extraction. Chia seed (*Salvia hispanica* L.) is widely cultivated in Latin America and Australia for its oils, and the revival of interest in chia seed is owing to its high oil content, providing a rich source of PUFAs [6]. The chia seed oil is unique since it contains the highest percentage of ω-3-linolenic acid (ALA) of any known vegetable source and other valuable constituents such as polyphenols, tocopherols, and phytosterols [7]. However, one major problem when using these types of sensible oils for human consumption is their high susceptibility to lipid oxidation. The process of oxidative deterioration of lipids by direct attack of carbon–carbon double bonds, especially in PUFAs, with free radicals in a process that is known as lipid peroxidation [8,9]. Lipid oxidation will generate harmful compounds such as aldehydes, ketones, and acids, thus resulting in undesirable off-flavors and leading to rancidity and nutrient loss, increasing the incidence of various diseases such as cancer, cardiovascular diseases, and age-related degenerative diseases, meanwhile causing huge economic loss [10].

Traditional methods for monitoring oil oxidation, such as peroxide value (PV), acid value (AV), and p-anisidine value (pAV), are well-established, but primarily quantify specific chemical endpoints, offering limited insight into the underlying oxidation process and physical and structural transformations. Usually, it is cumbersome to operate, time-consuming, requires the use of chemical reagents, and is a destructive test. It is reported that oil oxidation is fundamentally a free radical-mediated chain process, with the evolution of radical species serving as an indicator for assessing oxidative stability in oils. This underscores the inherent analytical attributes of electron spin resonance (ESR) spectroscopy-namely, exceptional sensitivity, rapid analysis, diagnostic efficacy, and direct measurement capabilities, which distinguish it from conventional methodologies. ESR probes the initiation stage of the oxidation chain reaction by directly quantifying radical species, which are the primary initiators of oxidation [8,11]. It is indispensable for studying oxidation mechanisms and evaluating the efficacy of radical-scavenging antioxidants, offering insights that are entirely inaccessible to chemical or other physical methods. ESR spin trapping, which is based on the formation of stable radicals (spin adducts) due to the reaction of free radicals and spin probes, has been widely employed to evaluate early stages of lipid oxidation in different food systems, such as the investigation of food oxidative stability, including different oils and oil adulteration [12,13,14].

Meanwhile, the oxidation of lipids is not only a complex chemical reaction process (finally generating hydroperoxides, aldehydes, ketones, acids, etc.) but also causes changes in the physical structure. Modern instrumental techniques such as low-field nuclear magnetic resonance (LF-NMR) and small-angle X-ray scattering (SAXS) offer complementary, non-destructive, and real-time monitoring capabilities, providing insights from fundamentally different perspectives. LF-NMR excels in monitoring the molecular dynamic changes, which are highly sensitive to changes that restrict molecular mobility, such as the formation of polymers and increased viscosity during early-stage oxidation. These physical alterations are often detected by LF-NMR as a change in the transverse relaxation time of hydrogen protons within the oil matrix well before a significant rise in traditional indices such as PV [15]. Conversely, SAXS can uniquely “visualize” the formation of oxidation-induced supramolecular assemblies created by polar products, which offers a mechanistic perspective and explains the physical consequences of oxidation (like visible sediment or turbidity) by revealing their structural origins at the nanoscale. It is exceptionally powerful for detecting the initial, minute aggregation of oxidation products in situ [16,17]. Therefore, in this work, we used the ESR spin-trapping technique combined with SAXS and LF-NMR to explore changes in radicals, physical structure, and molecular mobility involved in the oxidation process of chia seed oil.

## 2. Materials and Methods

### 2.1. Materials

Linoleic acid, linolenic acid, N-t-Butyl-α-phenylnitrone (PBN, 98%), and 5,5-dimethyl-1-pyrroline N-oxide (DMPO, 97%) were purchased from Sigma-Aldrich (St. Louis, MO, USA). Chia seeds were purchased from a local Walmart supermarket (Wuxi, China). All other chemicals used were of analytical grade and used as received.

### 2.2. Fatty Acid Composition Analysis of Chia Seed Oil

The oil was extracted from chia seeds using a hydraulic press (three-pillar Type A hydraulic oil press, Tianjin Sida Machinery Manufacturing Co., Ltd., Tianjin, China). Finally, the oil was centrifuged at 3000 r/min for 15 min, and the supernatant was taken and stored in the dark at 4 °C until use. The fatty acid composition of the chia seed oil was obtained on a GC-2010Plus gas chromatography (Shimadzu, Kyoto, Japan) using an Agilent SP-2560 capillary column (100 m × 0.25 mm × 0.2 μm). According to the AOCS Official Method Ce 1–62, 40 mg chia seed oil and 2 mL of 0.5 M NaOH-CH_3_OH were mixed and kept in a water bath at 60 °C for 30 min. Subsequently, 2 mL of 14% BF_3_-CH_3_OH reagent was added to the solution and kept at 70 °C for 10 min. 2 mL of hexane was added and shaken for 4 min, then 1.5 mL of the supernatant was taken, and anhydrous sodium sulfate was added for dehydration before centrifugation. For GC analysis, the oven temperature program was as follows: 70 °C (initial), increased to 140 °C at 50 °C/min, held for 1 min; raised to 180 °C at 4 °C/min, held for 1 min; then increased to 225 °C at 3 °C/min and held for 30 min. Inlet and detector temperatures were 250 °C and 300 °C, respectively. The injection volume was 1.0 μL, the flow rate of N_2_ was 1.0 mL/min with a split ratio of 45:1.

### 2.3. ESR Measurements

ESR measurements were performed using a Bruker EMXplus, X-band spectrometer (Bruker BioSpin GmbH & Co. KG, Ettlingen, Germany). Nitrone spin traps such as DMPO and PBN were separately used for the analysis of the oxidative stability of chia oil. Briefly, prior to in situ heating, 100 μL chia seed oil was added to the spin probe DMPO dissolved in toluene solution (0.1 M) and shaken well. Then the solution was placed into a quartz ESR sample tube with a 4 mm diameter and centered in the resonant cavity for ESR measurements. The PBN was directly added to the chia seed oil at a concentration of 0.3% (*w*/*w*), and the solution was used for the ESR test. The detailed instrumental parameters were as follows: sweep width 150 G, sweep time 30 s, center field 3350 G, modulation amplitude 1.0 G, modulation frequency 100 kHz, and microwave power 20 mW.

### 2.4. LF-NMR Analysis of Chia Seed Oil

The oil content of chia seed was measured by an LF-NMR analyzer (MesoMR23-060V-I, Niumag Co., Ltd., Suzhou, China). The magnetic field strength was 0.50 T, and the proton resonance frequency was 21 MHz. T_2_ relaxation measurements were performed based on the Carr–Purcell–Meiboom–Gill (CPMG) sequence, equipped with an inversion of a multiexponential fitting analysis program. 1 mL of oil was poured into a 2.0 mL threaded glass vial, followed by inserting the tube (15 mm in diameter) into the LF-NMR analyzer. The signal acquisition parameters were optimized and set as follows: TE (time echo) = 0.5 ms, TW (time waiting) = 4000 ms, NS (number of scans) = 2, NECH (number of echoes) = 8000.

### 2.5. SAXS Measurements

SAXS was used to monitor the lipid oxidation of chia seed oil. The oil was placed on a sample holder and sealed hermetically. Then, the sample was scanned using an Anton Paar SAXSpoint 2.0 system (Anton Paar, Graz, Austria) with a Microsource X-ray source, producing Cu Kα radiation at λ = 0.1542 nm. The SAXS data were collected for 30 min in the range of 0.06 < q < 4 nm^−1^, where q = 4π sin2θ/λ, with 2θ and λ representing the scattering angle and the X-ray wavelength, respectively. Data analysis was performed using self-configured software (SAXSanalysis 4.00.046) for the instrument.

### 2.6. Statistical Analysis

All experiments were conducted at least in triplicate. The data were presented as mean values ± standard deviation.

## 3. Results and Discussion

### 3.1. Fatty Acids Composition

**Scheme 1:**
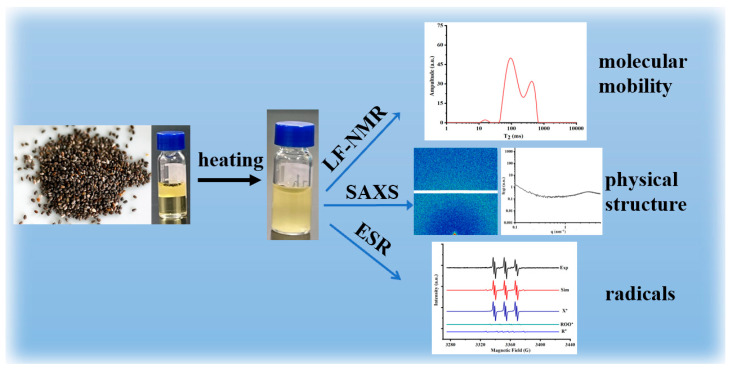
Schematic illustration for the study about the oxidation process of chia seed oil.

Chia seeds are renowned for their rich oil content, especially their high content of unsaturated fatty acids. In this paper, an oil content of about 35% was obtained by LF-NMR; the standard curve is displayed in Figure S1. However, this property brings about poor oxidative stability when chia seed oil is exposed to environmental factors such as air, light, and temperature, resulting in a shorter shelf life. Given the importance of edible oils, especially those highly rich in omega-3 polyunsaturated fatty acids, it is of great significance to evaluate the quality of chia seed oil. Despite the traditional methods that quantify specific chemical endpoints, we focus on the changes that occur during the oxidation process, such as changes in physical structure, radicals, and molecular mobility, as illustrated in Scheme 1.

Fatty acid composition is one of the most crucial factors representing the oxidative stability of edible oils [18]. Thus, to better understand the oxidation process, the fatty acid composition of the chia seed oil was initially determined by GC (Figure S2), and the fatty acid composition is shown in Table 1. A total of 10 fatty acids were presented, with linolenic acid (C18:3) as the most predominant fatty acid (63.03%), followed by linoleic acid (18.98%), oleic acid (7.88%), palmitic acid (6.18%), and stearic acid (3.37%), showing that PUFAs are predominated in chia seed oil, accounting for about 90%.

### 3.2. Study on Free Radicals of Chia Seed Oil by ESR

#### 3.2.1. Effect of Temperature

**Figure 1:**
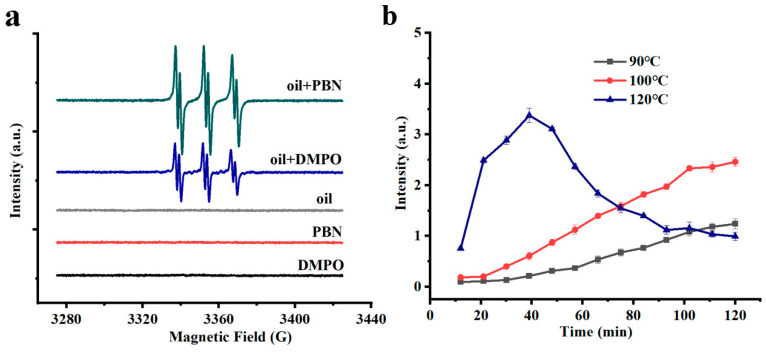
(**a**) ESR spectra of chia seed oil, (**b**) Change in radical intensity at different heating temperatures.

ESR spectroscopy was used to monitor the changes in radical intensity. ESR spin trapping, which is based on the reaction of radicals with diamagnetic molecules (spin probes such as PBN and DMPO) to form more stable radicals (spin adducts), was used to determine the oxidative process of chia seed oil. The ESR spectrum of chia seed oil is shown in Figure 1a. The control samples of oil, PBN, and DMPO solutions yielded negligible ESR signals, while very intense ESR signals were observed for oil heated at 100 °C, due to the generation of free radicals at high temperature. The effect of temperature on the ESR signal intensity was also conducted, as shown in Figure 1b. The intensity of free radicals increased as the heating temperature increased, and compared to the heating temperature of 90 °C, free radicals grew at 100 °C and 120 °C were more considerable. However, the free radicals grew rapidly and subsequently declined when heated at 120 °C. This might be attributed to the fact that, as the consumption rate of free radicals began to exceed their generation rate, the oxidation reaction reached a very intense level and entered a later stage dominated by the termination of free radicals and the formation of secondary products. Therefore, we selected 100 °C as the heating temperature in the following tests.

#### 3.2.2. Radicals Analysis of the Main Fatty Acids

**Figure 2:**
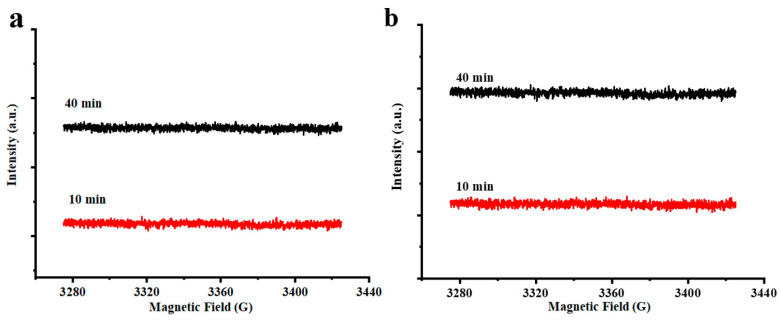
ESR spectra of saturated fatty acids. (**a**) palmitic acid, (**b**) stearic acid.

Based on the above GC results, we know that linolenic acid, linoleic acid, palmitic acid, and stearic acid were the most abundant unsaturated and saturated fatty acids, respectively. To better understand the changes in free radicals in the oxidation process of chia seed oil, ESR analysis of the individual lipid components following thermal treatment was subsequently studied. Figure 2 demonstrates that no obvious signal appeared in saturated fatty acids of palmitic acid and stearic acid. This might be ascribed to the heating energy that was insufficient to homogenize the stable C–C/C–H bonds and generate unpaired electrons [19,20]. Given the high content of unsaturated fatty acids, it is necessary to further replenish their free radical generation process for a better understanding of the main contribution to oil oxidation.

**Figure 3:**
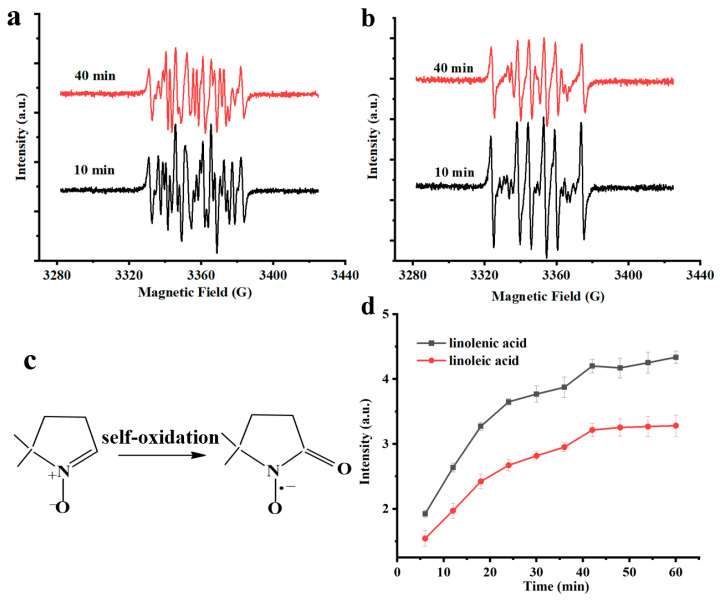
ESR spectra of unsaturated fatty acids: (**a**) linoleic acid, (**b**)linolenic acid, (**c**) self-oxidation of DMPO, and (**d**) the signal intensity of radicals with time.

It is reported that lipid oxidation proceeds via a free radical chain reaction, usually generating alkyl, peroxyl, and alkoxyl radicals. The process is initiated by the formation of alkyl radicals, which rapidly combine with oxygen to yield peroxyl radicals. These peroxyl radicals can further participate in reactions leading to alkoxyl radical formation. However, under different oxidation conditions and processes, the types of free radicals always varied [10,13,21]. To thoroughly investigate these radical species of linoleic acid and linolenic acid, DMPO as a spin-trapping agent was used to employ ESR analysis, and different heating times were selected to study the approximate changes (Figure 3a,b).

Both linoleic acid and linolenic acid exhibited characteristic sextet hyperfine splitting patterns in their ESR spectra. A comparative analysis of the signal profiles at different times; however, revealed that while the signal intensity was time-dependent, the overall spectral line shape remained invariant. This indicated that the identities of the dominant free radical species were conserved throughout the oxidation process. Thus, the experimental spectra of the samples were fitted by using a spinfitting function embedded in the Xenon software (Xenon 1.1b.81, Bruker) and three kinds of free radical species such as alkyl peroxy radicals (DMPO-•OOR), alkyl radicals (DMPO-•R), thought to be carbon-centered lipid-derived free radicals and DMPO oxidization radicals (DMPO-•X) (Figure 3c) are obtained for both linoleic acid and linolenic acid. In addition, alkoxy radical (DMPO-•OR) also existed in linoleic acid.

Overall, compared to the results of saturated fatty acids, the free radical intensity of linolenic acid and linoleic acid kept changing with the heating time. This is due to their highly unsaturated molecular structure that determines their extremely strong sensitivity to oxidation, enabling them to undergo efficient auto-oxidation chain reactions under thermal drive. This indicated that free radicals generated during the oxidation process originate primarily from unsaturated fatty acids [22]. Moreover, the spectral resolution for linolenic acid was more definitive at shorter heating durations, and this phenomenon was related to its oxidation rate [23]. Subsequently, PBN as a spin-trapping agent was used to study the overall growth of free radicals over time. As we can see from Figure 3d, the increase rate of linolenic acid was even faster than that of linoleic acid. This is in line with the classical theory that the relative self-oxidation rate of fatty acids is directly proportional to the number of active methylene groups in their molecules [24].

#### 3.2.3. Analysis of the Radicals in Chia Seed Oil

**Figure 4:**
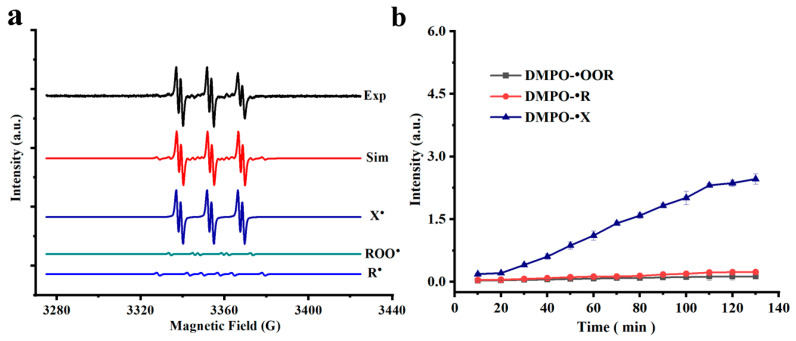
(**a**) Experimental and simulated ESR spectrum of chia seed oil (Exp, experimental spectrum; Sim, computer simulation of the experimental spectrum; ROO•, simulation of the DMPO-•OOR spectrum; R•, simulation of the DMPO-•R spectrum; X•, simulation of DMPO oxidization radicals; (**b**) The signal intensities of alkyl, peroxyl and DMPO oxidization radicals in the chia seed oil sample.

Regarding the study on free radical signal changes in individual saturated fatty acids and unsaturated fatty acids, DMPO was used as the spin agent, and the heating condition was 100 °C for the analysis of species of lipid-derived free radicals in chia seed oil. Based on the hyperfine coupling splitting constants of different free radicals, three major radical adducts were observed, namely DMPO-•R, DMPO-•OOR, and DMPO-•X (Figure 4a). Different from some existing studies [8,13], alkoxyl radical was not observed here; the failure to detect alkoxyl radical adducts may not indicate their absence in the oxidation process but rather could be a direct consequence of their secondary formation, exceptionally high reactivity, and short lifetime, which collectively maintain their steady-state concentration below the threshold of ESR detection.

In contrast, the peroxyl radical, as the key chain-carrier with a relatively longer lifetime (millisecond scale), and the alkyl radical, especially under transient oxygen-limiting conditions, achieve sufficiently high concentrations to form stable, detectable adducts with DMPO. Figure 4b illustrates the temporal evolution of three distinct free radical species generated during the oxidation of chia seed oil at 100 °C. The ESR analysis identified the DMPO-•X as the predominant species. In contrast, the concentrations of the DMPO-•R and DMPO-•OOR remained consistently low throughout the heating period. These results indicate a steady progression of oil oxidation at 100 °C.

### 3.3. LF-NMR Study of Chia Seed Oil

**Figure 5:**
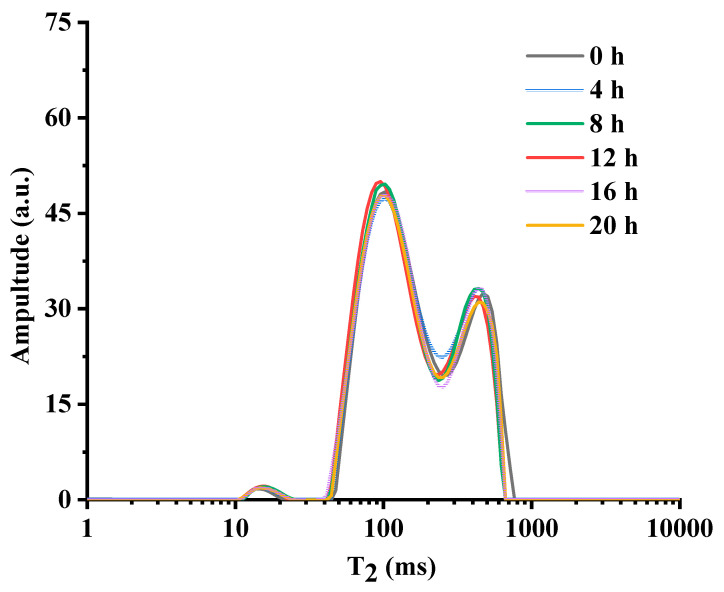
The T_2_ distribution of chia seed oil heated at different times.

Generally, hydrogen protons at different structural positions in the same molecule also have different T_2_ [25]. Thus, by tracking changes in the molecular dynamics of the oil system, as reflected by T_2_ of hydrogen protons, we can monitor lipid oxidation. The data of LF-NMR echo decay curves are collected and inverted to obtain the multi-component relaxation profiles of the oil under different heating times, as displayed in Figure 5. A trimodal distribution with three peaks at T_21_ (10–20 ms), T_22_ (80–100 ms), and T_23_ (300–800 ms) was exhibited in their respective relaxation spectra, and their peak areas were noted as S_21_, S_22_, and S_23_. This is consistent with the results from Hu’s report [26], where three characteristic peaks were observed in the multi-component relaxation profile of 9 edible oil samples.

Figure 5 exhibits that with the prolongation of heating time, the multi-component relaxation profile of the chia seed oil showed an overall left-shifting trend (in the direction of shorter relaxation time). That is, T_21_, T_22_, and T_23_ decreased gradually together with their corresponding S_23_ and S_22_, which had some fluctuation, but S21 presented a slightly increasing trend, and the decrease trend of S_23_ was relatively more obvious than that of S_22_. This may be due to the occurrence of many complex chemical reactions of unsaturated fatty acids during heating. As oxidation progresses, it induces the formation of polymerization products and polar compounds, which increase the system’s viscosity and strengthen intermolecular interactions. These physical and chemical alterations restricted the mobility of hydrogen protons within the magnetic field, consequently accelerating their relaxation rate and leading to a measurable decrease in the T_2_ relaxation time and an overall leftward shift in the relaxation spectrum [15,25,27]. On the other hand, the fragmentation of peroxides into volatile low-molecular-weight species such as aldehydes and ketones, and the concurrent accumulation of polymeric compounds, promoted this phenomenon. This is consistent with the results from Wang [28] et al., Zhao [29] et al., and Sun [30] et al., where camellia seed oil, palm oil, and soybean oil were, respectively, used for frying.

### 3.4. SAXS Analysis of Chia Seed Oil

**Figure 6:**
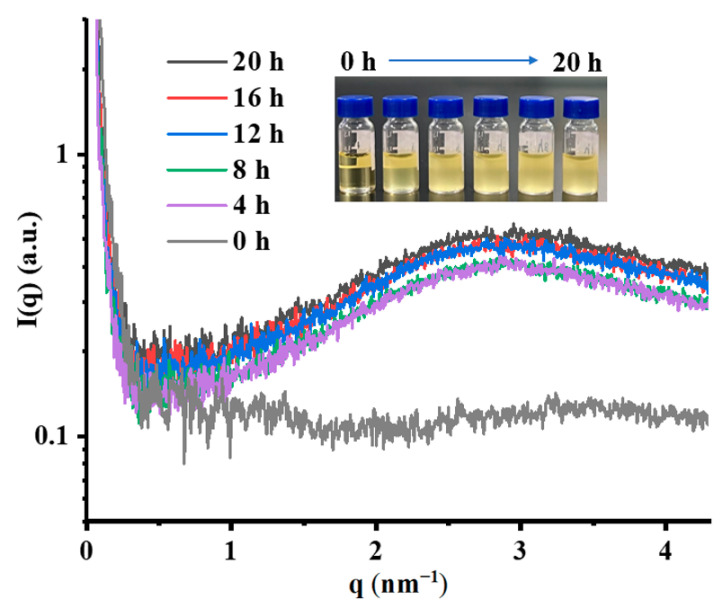
Scattering curves of chia seed oils with different heating times. The insert shows the corresponding photograph.

Lipid oxidation constitutes not only a complex chemical process (generating hydroperoxides, aldehydes, ketones, acids, etc.) but also induces alterations in the physical structure of lipids. SAXS analysis technology, being highly sensitive to variations in electron density, is uniquely capable of capturing this structural evolution [16,31]. Thus, SAXS was employed to monitor the nanostructural evolution of chia seed oil throughout its oxidation pathway. As shown in Figure 6, only a weak, broad, and diffuse peak appeared around q = 3.4 nm^−1^ (i.e., d = 1.8 nm, 2π/q_peak_), which corresponded to the average correlation distance between polar parts of triglycerides [32]. This demonstrated that fresh chia seed oil did not display any organization, and the relatively flat and low-intensity scattering curve indicated a mainly homogeneous system at the nanoscale with an essentially single-phase structure devoid of significant nanoscale features or electron density fluctuations. As the oil underwent thermal treatment, distinct changes emerged. Namely, the scattering intensity in the entire region exhibited a pronounced increase, developing a diffuse peak feature at q = 2.8 nm^−1^ (i.e., d = 2.2 nm, 2π/q_peak_). This intensified scattering evolved progressively with extended oxidation time, indicating that as the heating proceeded, nano-aggregates were formed, and these nano-aggregates were not of a single size [33].

In addition, compared to fresh chia seed oil, the thermal treatment slightly shifted the scattering peak to the low q-region. The enhancement in intensity and peak shift phenomenon may directly correlate with the development of nanoscale heterogeneities, suggesting the formation and growth of aggregated phases derived from oxidation products. This was consistent with the appearance of the oil we observed, which showed that the turbidity of the chia seed oil gradually deepened as the heating time increased, and our findings from LF-NMR were complemented with the SAXS data [34]. In summary, the coexistence of a diffuse peak in the SAXS curve and visible changes in turbidity provided direct evidence for the formation of polydisperse nanoscale aggregates with random orientation during oil oxidation.

## 4. Conclusions

To summarize, we established a multi-technique protocol based on electron spin resonance, low-field nuclear magnetic resonance, and small-angle X-ray scattering for studying the oxidation process of chia seed oil. By the ESR method, three main free radicals were generated in the accelerated oxidation of chia seed oil. This approach captured the precursor events of chia seed oil degradation, which are undetectable by conventional analytical methods. Meanwhile, transverse relaxation parameters by LF-NMR and SAXS results identified oxidation products of macromolecules that were generated within the system, which directly contributed to observable physical changes in the oil. Considering the important roles of the oils, especially those rich in omega-3 polyunsaturated fatty acids, such a method can likely provide a predictive platform for assessing the oxidative stability of oils.

## Figures and Tables

**Table 1:** Fatty acid composition of chia seed oil.

Fatty Acids	Content (%)
Myristic acid (14:0)	0.03 ± 0.01
palmitic acid (16:0)	6.18 ± 0.02
stearic acid (18:0)	3.37 ± 0.08
Arachidic (20:0)	0.26 ± 0.00
Behenic acid (22:0)	0.08 ± 0.01
Palmitoleic acid (16:1)	0.07 ± 0.03
Oleic acid (18:1)	7.88 ± 0.05
Eicosenoic acid (20:1)	0.13 ± 0.01
Linoleic acid (18:2)	18.98 ± 0.09
Linolenic acid (18:3)	63.03 ± 0.03

## Data Availability

The original contributions presented in this study are included in the article/Supplementary Materials. Further inquiries can be directed to the corresponding author.

## Supplementary Materials

The following supporting information can be downloaded at: https://www.mdpi.com/article/10.3390/foods14244280/s1. Figure S1: GC spectrum of chia seed oil; Figure S2. Standard curve for oil content.

## Abbreviations

The following abbreviations are used in this manuscript:

LF-NMR	Low-field nuclear magnetic resonance
SAXS	Small-angle X-ray scattering
ESR	Electron spin resonance
