# Supplementary figures and images for: Evaluation of the Oxidative Process of Chia Seed Oil by Means of ESR Combined with LF-NMR and SAXS
Source: Foods. 2025 Dec 12;14(24):4280. doi: 10.3390/foods14244280 (PMC12732257; doi:10.3390/foods14244280)

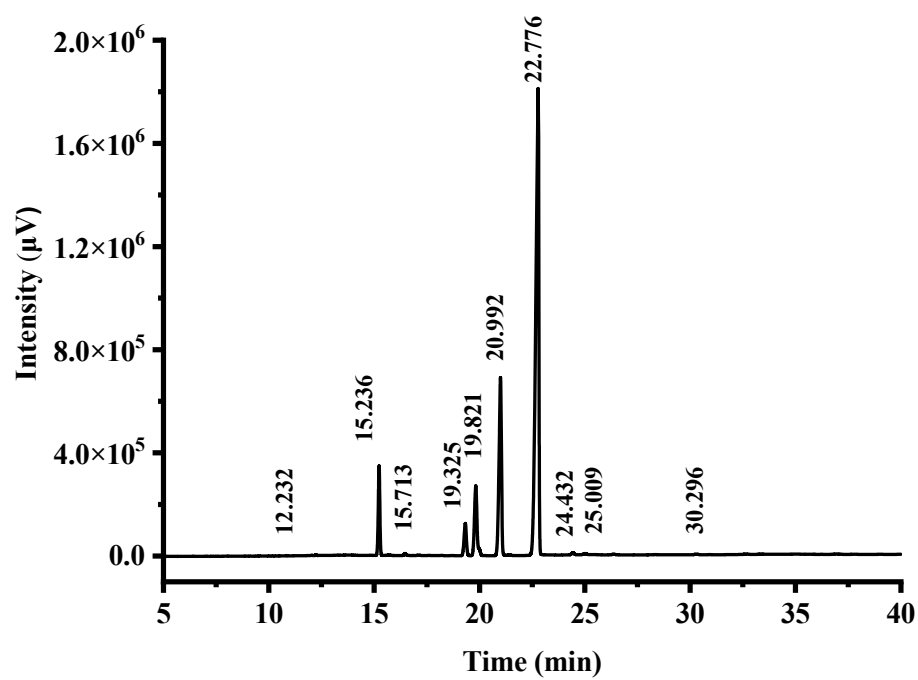

Figure S1. GC spectrum of chia seed oil.

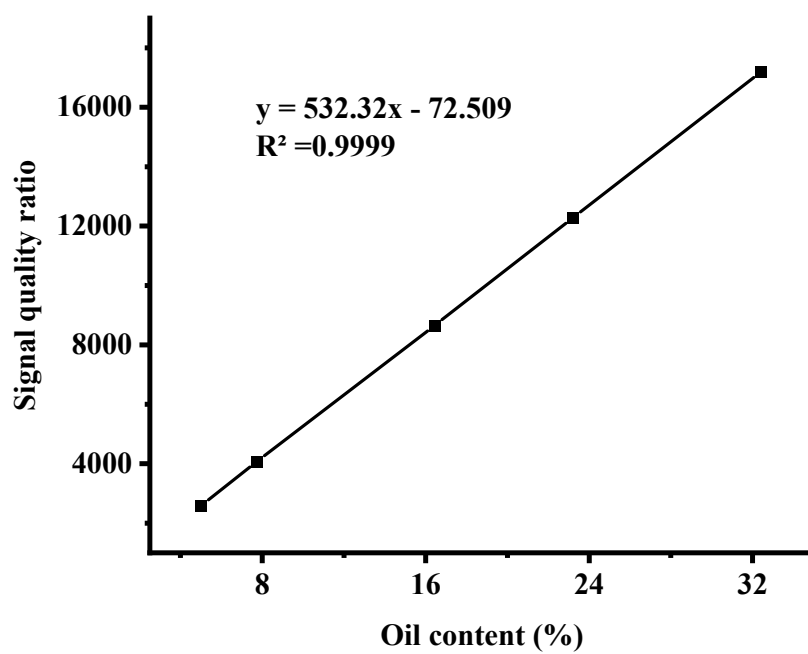

Figure S2. Standard curve for oil content.

Supplement: Supplementary file 1 [file foods-14-04280-s001.zip › foods-3984409-supplementary.pdf]
